# Cost-effectiveness analysis of two thromboprophylactic strategies following major surgery

**DOI:** 10.1186/cc11029

**Published:** 2012-03-20

**Authors:** C Ebm, M Cecconi, A Rhodes, M Grounds

**Affiliations:** 1 St George's Healthcare Trust, London, UK

## Introduction

Patients recovering from major surgery are at high risk of developing life-threatening deep venous thrombosis, which is a key source of postoperative morbidity and mortality. Our objective was to assess the cost-effectiveness of two different thromboprophylactic agents for patients admitted to the ICU after high-risk surgery: intermittent pneumatic compression (IPC) and anti-embolism stockings (AES).

## Methods

A decision model (TreeAge Software 2010) was constructed simulating the impact of AES and IPS on patient outcomes and costs following high-risk surgery in the UK. Probabilities were assessed from published data [1]. ICU and item costs were derived from NHS reference costs tablets. Assessed outcomes were cost per deep vein thrombosis (DVT) and pulmonary embolism (PE) prevented, net monetary benefit and incremental costs per quality-adjusted life expectancy (QALY) gained.

## Results

Total costs for in-patients receiving AES were £923 and £1,010 for patients treated with IPC. Equipment costs and cost of initial care were higher in patients who received IPC, but this was partly offset by a reduction in costs related to treatment of early (DVT and PE) and late complications (post-thrombotic syndrome and pulmonary hypertension). IPC treatment increased QALY by approximately 0.01 years. The incremental cost-effectiveness of the IPC device was £12,650 per QALY gained. One-way sensitivity analysis revealed that the most sensitive variables were probability of developing a DVT resulting from the insignificant difference in treatment efficacy.

## Conclusion

Based on UK cost-effectiveness guidelines, our results indicate that IPC stockings should be used for patients at high risk of developing DVT. IPCs decrease the incidence of developing DVT and therefore result in cost savings related to preventive and therapeutic actions. For patients at low risk of developing DVT, AES are favoured due to higher utility and lower maintenance costs associated with AES. Due to the lack of reliable data on the incidence of PE as well as the absence of reliable head-to-head studies between IPC and AES, no generalisable conclusion to favour either strategy can be made.
